# Comparison between ultrasound guided erector spinae plane block and paravertebral block on acute and chronic post mastectomy pain after modified radical mastectomy: randomized controlled trial

**DOI:** 10.1186/s12871-024-02810-4

**Published:** 2024-11-21

**Authors:** Samy Abdelrahman Amr, Ahmed Hassan Othman, Eman Hassan Ahmed, Romany Gergis Naeem, Shereen Mamdouh Kamal

**Affiliations:** 1 Anesthesia, Intensive Care and Pain Management Department, Faculty of Medicine, Assiut University, Assiut, Egypt; 2 Anesthesia, Intensive Care and Pain Management Department, South Egypt Cancer institute, Assiut university, Assiut, Egypt; 3 Clinical Pathology South Egypt Cancer Institute Assiut University, Assiut, Egypt

**Keywords:** Postmastectomy pain, Erector spinae block, Paravertebral block

## Abstract

**Background:**

Inadequate acute postoperative pain management is linked to the effect on the stress response and development of chronic pain. A unique regional anaesthetic method that is becoming more important for postoperative pain management is erector spinea plane block (ESP). Since its initial description, physicians have questioned weather this novel easy method can take the place of paravertebral block (PVB). Our goal was to evaluate, in contrast to control group, the effects of ESP & PVB on acute and chronic post-mastectomy pain.

**Methods:**

One hundred and five female patients undergoing modified radical mastectomy participated in this study, randomly allocated into three equal groups: erector spinae plane block (ESP), thoracic paravertebral (TPV), and control groups. Both blocks were ultrasound-guided with 20 ml 0.25% bupivacaine according to patients’ group, control group was administered standard general anaesthesia without intervention. Total morphine consumption in the first 24 h postoperative was the primary outcome. The secondary outcomes were time to the first analgesia, (Visual Analogue Scale)VAS score, serum level of cortisol and prolactin, sedation score, side effects, and LANSS scores in the first, third, and sixth postoperative months were among the variables compared between groups.

**Results:**

Total morphine consumption in the first 24 h was significantly higher in control than ESP and TPV groups (10.74 ± 1.37, 8.17 ± 1.69, 5.70 ± 1.95 respectively *p* < 0.001). Time to first analgesic request was the shortest in control versus ESP and TPV groups as (4.37 ± 3.06, 8.13 ± 1.75, 10.64 ± 1.83 h respectively p ˂0.001). ESP and TPV had significantly lower cortisol and prolactin levels compared to control (*p* < 0.001). The highest LANSS scores were in the control group in the first, third, and sixth months compared with ESP and TPV, with no significant difference between ESP and TPV.

**Conclusion:**

ESP and TPV blocks provided superior early postoperative analgesia and reduced stress response compared to the control group in female patients undergoing modified radical mastectomy. PVB is better than ESB in acute postoperative pain management (the total morphine consumption VAS score and time of first analgesic request). Both techniques showed better long-term outcomes compared to the control group regarding LANSS score in the 6-month follow-up.

**Trial registration:**

https://www.ClinicalTrials.gov trial registry (identifier NCT04498234 on 04/08/2020).

**Supplementary Information:**

The online version contains supplementary material available at 10.1186/s12871-024-02810-4.

## Introduction

Among women, breast cancer is the most common cancer. Given the high frequency of breast cancer, one of the most common surgical procedures is the mastectomy. Because breast surgery is complex and the breast has a complex nerve system, analgesia after the procedure might be problematic [1].

Acute postoperative pain that is poorly managed is thought to be another risk indicator for a subsequent occurrence of chronic pain [2]. It causes persistent pain and hyperalgesia by inducing central sensitization.

After surgery, chronic pain affects 30% of patients, impairs function and quality of life, induces distress, and is sometimes irreversible and challenging to manage. It is now much more important to prevent chronic pain than to treat it. Central sensitization is a comprehensive stepwise process and so, the integration of nociceptive impulses over time leads to persistent postoperative pain [3]. Blocking nociception during any part of the perioperative experience may prevent persistent pain after surgery, raising the importance of pre-emptive or even preventive regional analgesic techniques [4].

Although opioids are commonly used to treat acute post-mastectomy pain, they can have several negative side effects, including nausea, vomiting, and respiratory depression.

There is evidence that using regional anaesthesia techniques during breast cancer surgery could potentially reduce the surgical stress response and indirectly aid in tumor inhibition by reducing the use of opioids, which have been associated with immunosuppression and progression of cancer [5].

Even though thoracic epidural analgesia and paravertebral block (PVB) have been linked to significant complications such as pneumothorax and total spinal anesthesia, they have become the gold standard for breast surgery. Because of the use of ultrasound in regional anesthesia, various novel blocks have been developed that can give analgesia for breast procedures with fewer adverse effects [6].

The erector spinae (ES) is a combination of three muscular layers: the iliocostalis, longissimus, and spinalis. These layers extend from the lower back base of the skull superiorly down to the pelvis, running parallel to each other across the vertebra. There is a space deep to the ES muscle, the ES facial plane, which runs from the nuchal fascia cranially to the sacrum caudally (C7 - T2 cranially and L2 - L3 caudally), where the injected local anesthetic (LA) diffuses cranio-caudally for many levels. LA effects block the dorsal rami of spinal neurons [7–9]. We tested the hypothesis that ESP block could lower the total amount of morphine taken by having an opioid sparing effect. It also offers comparable early postoperative analgesia to PVB for breast surgeries and lowers development of chronic pain later.

Our primary aim was to assess the analgesic efficacy of erector spinea muscle block and paravertebral block in comparison to control group in patients scheduled for elective unilateral breast surgery regarding total morphine consumption in the first 24 h postoperative. The secondary aims were time of first request of analgesia, pain scores, patient’s sedation, patient’s satisfaction, serum cortisol and prolactin levels, LANSS score at the 1st, 3rd, 6th months postoperative.

## Patients and methods

### Study design and ethics

This prospective randomized controlled study was approved by the Research Ethics Committee of the South Egypt Cancer Institute, Assiut University, Assuit, Egypt (SECI-IRB/IORG0006563/Approval No. 497). Our protocol strictly followed the regulations and amendments of the Helsinki Declaration and was prospectively registered in the ClinicalTrials.gov trial registry (identifier: NCT04498234 on 04/08/2020). The study was conducted according to the Consolidated Standards of Reporting Trials (CONSORT) statement. All study participants provided their written informed consent.

### Participants

One hundred and five female patients between the ages of 20 and 70 were enrolled and scheduled for either a right or left-modified radical mastectomy, with an American Society of Anaesthesiologists (ASA) physical status of I or II. Females with ASA III; those who refused to participate; who had coagulopathy; drug hypersensitivity, or allergy to the drugs under study; those with central or peripheral neuropathy; who had consumed opioids within 24 h before surgery; with abnormalities of the vertebral column; who had suffered a stroke; or who had a mental illness that could impact pain perception, were excluded from the study.

### Outcomes

**The primary outcome** was a comparison of total morphine consumption during the first 24 h postoperatively among those who had received either an ultrasound-guided thoracic paravertebral block or an ultrasound-guided Erector Spinae plane block and the control group.

**The secondary outcomes were**:

- The effect of these blocks on the Visual Analogue Score (VAS) [10] during the first 24 h postoperatively, both at rest and during movement (abduction of the ipsilateral arm).
- The time of the first request for analgesia.
- The postoperative vital signs.
- The stress response to surgery and anesthesia (obtained by measuring cortisone and prolactin levels in serum).
- The level of sedation in accordance with the Modified Observer’s Assessment of Alertness/Sedation Scale (MOAA/S) [11].
- Satisfaction levels.
- Side effects.
- Chronic post-mastectomy pain at the first, third, and sixth months postoperatively in terms of the Leeds Assessment of Neuropathic Symptoms and Signs (LANSS) score [12].

### Randomization and blinding

Randomization was done with a computer-generated randomization algorithm (http://www.randoiler.org), according to which the participating females were divided into three groups. In the control (C) group: (35 patients) general anesthesia was administered to the patients according to the standard protocol. In the erector spinae block (ESP) group (35 patients), general anesthesia was administered in combination with an erector spinae block, and in the thoracic paravertebral (TPV) group (35 patients), and general anesthesia was administered in combination with a thoracic paravertebral block.

The attending anesthesiologist, the patients, and the data-collecting staff were all blinded to the research group assignment of the patient. However, the investigator who carried out the blocks was not.

### Procedures

After the fasting period had passed, standard monitors (ECG, non-invasive blood pressure, pulse oximetry, and capnography) were applied and recorded every 5 min until completion of the operation, and an intravenous 18-gauge cannula was introduced and secured. Before surgery, the first blood sample of 5 mL of venous blood was collected to measure the preoperative serum levels of prolactin and cortisol. Patients in the control group (C) were given anesthesia according to the standard protocol.

**Fig. 1:**
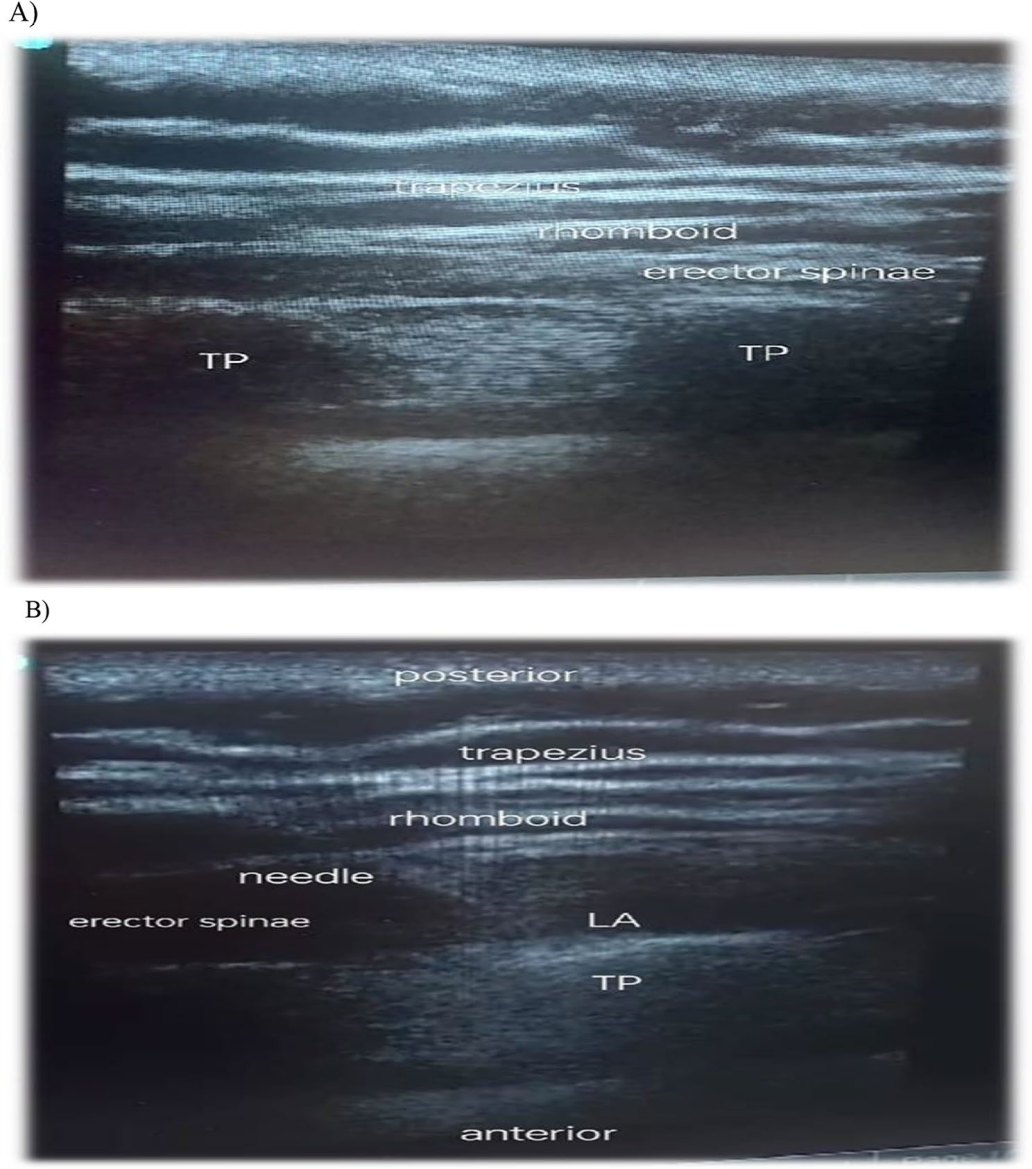
Ultrasound image of erector spinae plane block pre and post technique. **A**) pre-block. TP: Transverse process, **B**) post-block image. TP: Transverse process, LA: Local anaesthetic

The (ESPB) group patients had an ultrasound-guided erector spinae block while seated, with the upper back skin prepped with 2% chlorhexidine solution, depending on the surgery location (Lt or Rt). After identifying the trapezius muscle, the rhomboideus major muscle, and the erector spinae muscle from the surface, an ESP block using a high-frequency linear U/S transducer was applied. The probe was positioned longitudinally lateral to T4, and lateral to the spinous procedure to visualize the transverse process. Next, an in-plane needling technique was used to deposit a local anesthetic, 0.25% bupivacaine (20 mL) slowly at the level of T4 into the interfacial plane below the erector spinae muscle (Fig. 1).

**Fig. 2:**
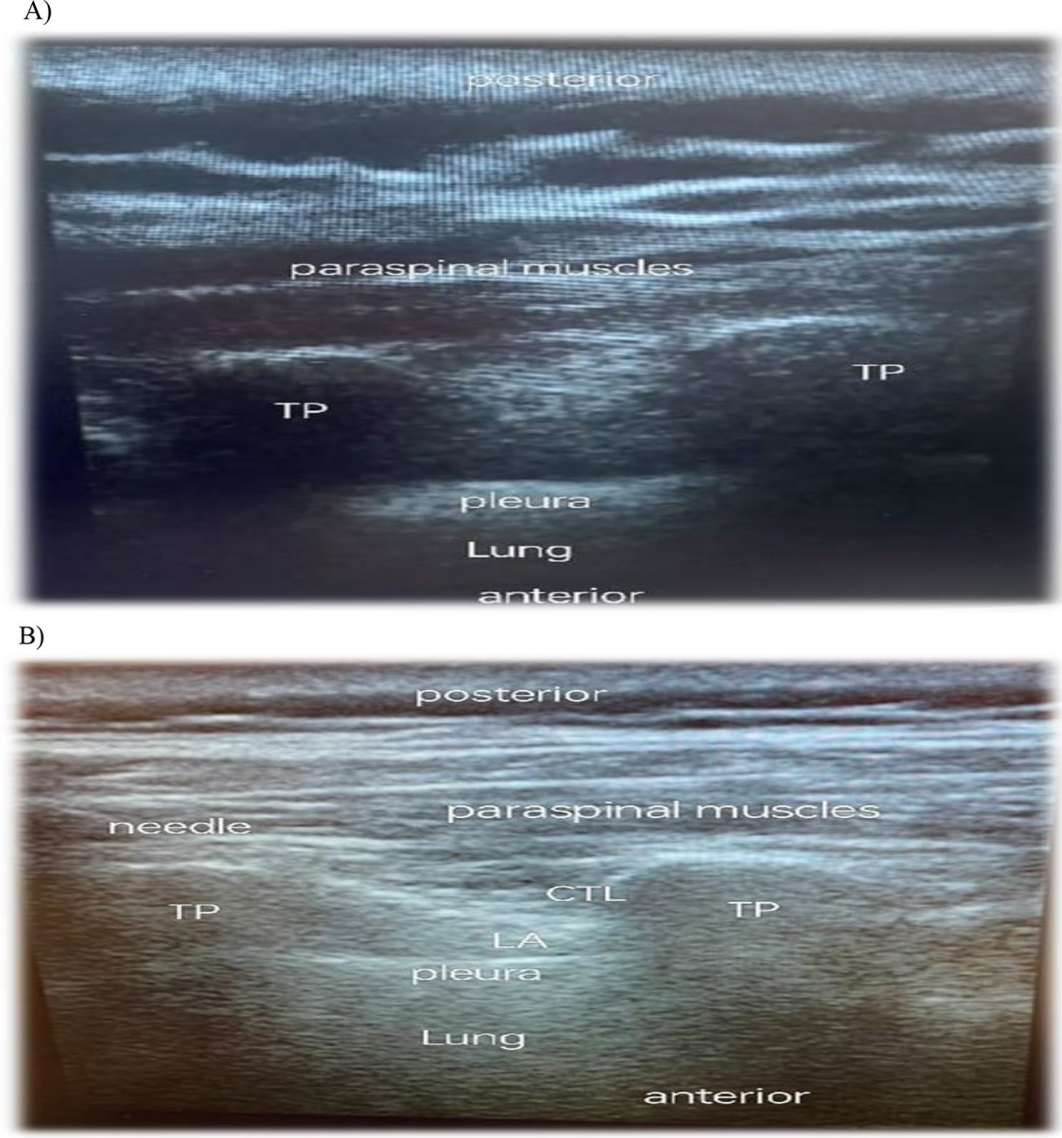
Ultrasound image of thoracic paravertebral block pre and post technique. **A**) pre-block image. TP: Transverse process, **B**) post-block image. TP: Transverse process. CTL: Costotransverse ligament, LA: Local an aesthetic

An ultrasound-guided thoracic paravertebral block was administered to TPVB group patients while they were seated and in accordance with whether the surgical location was left or right. The skin of the upper back was prepped with 2% chlorhexidine solution. The high-frequency linear U/S transducer utilized for TPV was positioned at the T2, T4, and T6 levels, parallel to the spinal column, to ensure adequate vision. The ultrasound probe was adjusted 2–3 cm laterally. After the pleura, transverse process, and paravertebral space were identified, an in-plane technique was used to implant the needle in the caudo-cranial direction. No pleural or negative vascular breach was caused by aspirating. Then 0.25% bupivacaine (20 mL), was gently administered at T2, T4, and T6. The pleura were pushed lower during this procedure (Fig. 2).

In all the studied patients, after the establishment of the TPVB or ESPB, anesthesia and muscle relaxation were induced with fentanyl 0.5 µg/kg, propofol 2 mg/kg, and atracurium 0.5 mg/kg. These were used to induce and facilitate intubation and the induction of general anesthesia. Following a patient’s intubation, a second blood sample was withdrawn. The anesthesia was maintained by the inhalation of 1–1.5 MAC of isoflurane, and oxygen-and-air mixes to compose an inspired oxygen fraction (FIO2) of 0.5. Furthermore, frequent muscle relaxants were administered every 20 min. Patients were mechanically ventilated to maintain an end-tidal (ETCO2) pressure of between 35 and 40 mmHg. At the end of the operation, safe extubation was performed after the administration of a reverse muscle relaxant (0.05 mg/kg of neostigmine and 1 mg of atropine).

### Outcome assessment and data collection

The postoperative care unit monitored all patients for the first 24 h following surgery. Hence, they were assessed for their serum cortisol and prolactin levels immediately after surgery (the third sample) and 24 h later (the fourth sample). Their vital signs and VAS scores, measuring pain intensity at rest and during movement (abduction of the ipsilateral arm at 90 degrees), were monitored immediately after surgery and at 2, 4, 6, 12, and 24 h postoperatively. The first request for intravenous morphine analgesia following surgery and the total amount of morphine used in the first 24 h following surgery were noted. The IV-PCA solution contained 100 mg morphine in 50 mL 0.9% normal saline (2 mg/mL). The IV-PCA program consisted of an initial morphine bolus of 2 mg when pain was expressed, or if VAS ≥ 3. A lockout period of 5 min with no background infusion was allowed. Alertness/sedation scale assessments, patient satisfaction levels, and the occurrence of postoperative side effects such as itching, nausea, and vomiting were recorded and assessed. Patients were evaluated in the pain clinic during the first, third, and sixth months after surgery using a LANSS score to detect the occurrence or absence of chronic pain.

### Laboratory assessment

Venous blood (5 mL) was withdrawn to a newly designed, high-sensitivity Elecsys analyzer (totally automated ELISA EVOLIS BIROAD, France), which was used to assess cortisone and prolactin concentrations in all of the patients preoperatively (T0), immediately after intubation (T1), immediately after extubation (T2), and during the 24-h postoperative (T3) times. The samples were centrifuged and stored at -70ºC.

### Data analysis

#### Sample size calculation

G*Power 3 software 1 was used to compute the sample size. A minimum of 102 women who were candidates for mastectomy surgery were randomly allocated to one of three equal groups (*n* = 34).To detect an effect size of 0.2 in the ANOVA for the mean total morphine consumption in the first 24 h postoperatively with an error probability of 0.05 and 90% power, the control group will get a standard regimen of anaesthesia, the ESP group will receive an erector spinae plane block, and the TPV group will receive a thoracic paravertebral block [13, 14]. Considering potential drop-outs, we decided to enroll 35 patients in each group for the study.

### Statistical analysis

SPSS (Statistical Package for the Social Science, version 20, IBM, Armonk, New York) was used to gather and analyze the data. To ascertain if the data adhered to a normal distribution, the Shapiro test was employed. ANOVA was used to examine quantitative data, which were represented as mean ± standard deviation (SD), and a post-hoc test was performed after. Number (n) and percentage (%) are used to represent nominal data. Chi2 testing was used for these kinds of data. Because the 95% level of confidence was maintained, a P value was deemed significant if it was less than 0.05.

## Results

### Patients’ characteristics

**Fig. 3:**
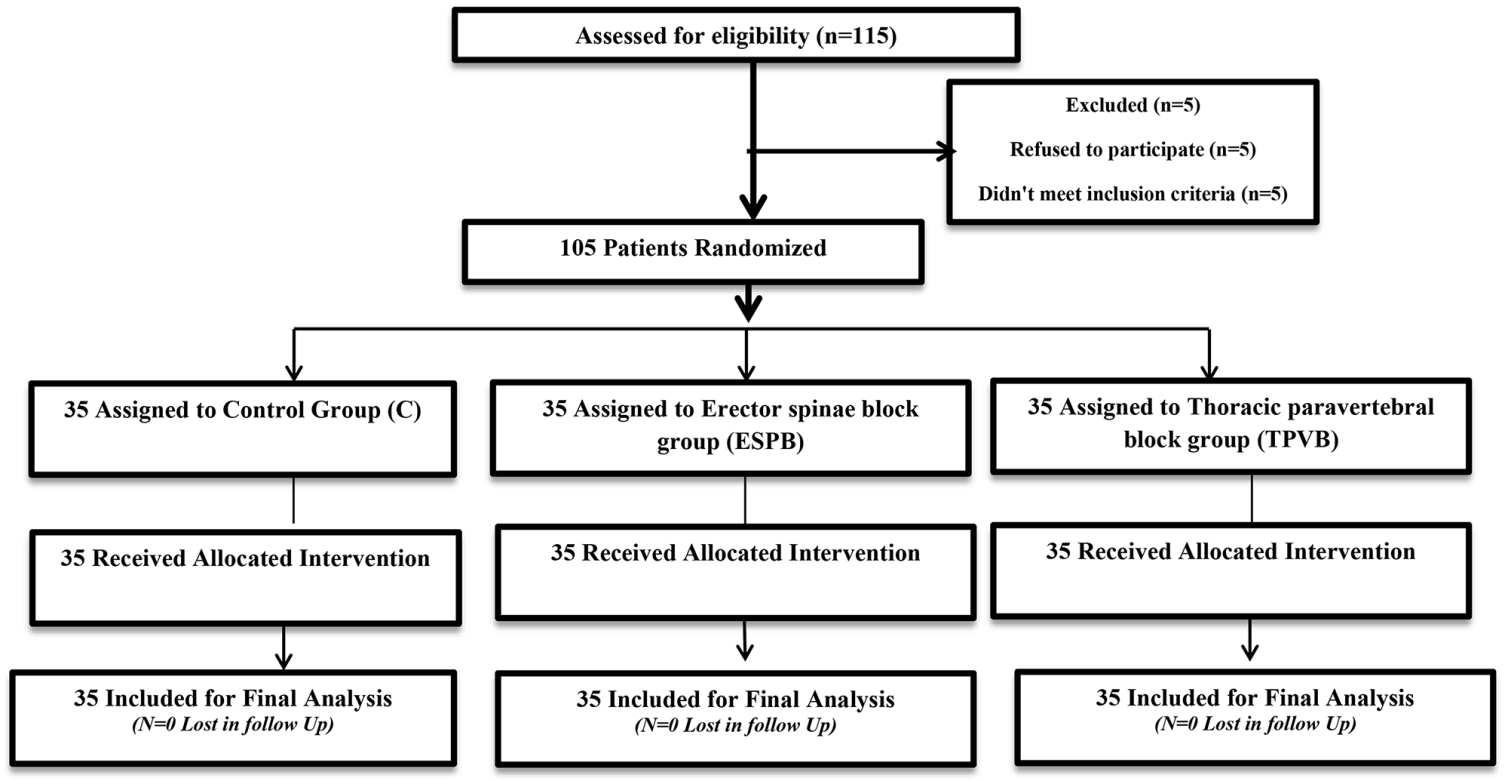
Consort flow diagram of the study participating patients

One hundred and five patients, randomized into three equal groups (*n* = 35 for each group), completed this study, and were subjected to statistical analysis, as shown in Fig. 3. No statistical differences were observed among the three studied groups regarding age, Body Mass Index (BMI), ASA physical state, duration of anesthesia, duration of surgery, and site of surgery (p-value ˃ 0.05) (Table 1).

**Table 1:** Patients’ demographic and clinical data

	ESP bock (*n* = 35)	TPV block (*n* = 35)	Control group (*n* = 35)	*P* value	P1	P2	P3
Age (years)	43.63 ± 9.89	42 ± 8.71	45.49 ± 9.73	0.30	0.47	0.41	0.0.12
weight	72.13 ± 7.65	72.67 ± 7.59	73.13 ± 7.41	0.808	0.89	0.18	0.09
Height	165.27 ± 5.13	164.53 ± 5.23	163.93 ± 5.13	0.599	0.41	0.30	0.32
BMI	26.56 ± 3.93	27.00 ± 3.97	27.36 ± 3.91	0.709	01.7	0.20	0.18
Site of operation R/L	16/19	17/18	19/16	0.765	0.81	0.47	0.63
ASA class				0.68	0.06	---	0.06
Class-I	24 (68.6%)	21 (60%)	24 (68.6%)				
Class-II	11 (31.4%)	14 (40%)	11 (31.4%)				
Duration of anesthesia (minute)	112.97 ± 11.43	114.46 ± 11.69	112.09 ± 14.36	0.69	0.57	0.77	0.39
Duration of surgery (minute)	96.09 ± 10.65	98.54 ± 11.28	96.69 ± 12.57	0.65	0.37	0.82	0.50

Data expressed as mean (SD), frequency (percentage). *P* value was significant if < 0.05. **ESP**: erector spinae plane block; **TPV**: thoracic paravertebral block; **ASA**: American society of anesthesiologists, **BMI**: Body Mass Index

***P*value** compares between different groups

***P*1** compares between ESP and TPV groups

***P*2** compares between ESP mg and control groups

***P*3** compares between TPV mg and control groups

*Analysis was done by ANOVA test followed by post-hoc analysis for continuous data otherwise Chi^2^ test was used for analysis

### Study endpoints

#### Primary outcome

According to the total morphine consumption in the first 24 h postoperatively, ESP and TPV were significantly lower in comparison to the control group (*p* < 0.001), and ESP was significantly higher in comparison to the TPV block (*p* < 0.001) (Table 2).

**Table 2:** Postoperative VAS and analgesic consumption among the studied groups

	ESP bock (*n* = 35)	TPV block (*n* = 35)	Control (*n* = 35)	*P* value*	Subgroup analysis		
					P1	P2	P3
**Time to 1st analgesia (hr)**	8.13 ± 1.75	10.64 ± 1.8	4.67 ± 3.1	< 0.001	0.03	< 0.001	< 0.001
**Total analgesic consumption (mg)**	8.17 ± 1.7	5.70 ± 1.9	10.74 ± 1.4	< 0.001	< 0.001	< 0.001	< 0.001
**Number of Morphine Bolus**				< 0.001			
•→ **2**	1 (2.9%)	5 (14.3%)	0 (0%)				
•→ **3**	1 (2.9%)	3 (8.6%)	0 (0%)				
•→ **4**	6 (17.1%)	2 (5.7%)	3 (6.6%)				
•→ **5**	4 (11.4%)	0 (0%)	17 (48.6%)				
•→ **6**	0 (0%)	0 (0%)	15 (42.9%)				
**Number of patients requested analgesia**	12	11	35	< 0.001	0.09	< 0.001	< 0.001
**VAS (at rest)**							
Immediately	3.14 ± 0.49	3.30 ± 0.50	5.83 ± 0.17	< 0.001	0.72	< 0.001	< 0.001
2nd hr	3.74 ± 0.93	3.34 ± 0.62	7.31 ± 0.36	< 0.001	0.31	< 0.001	< 0.001
4th hr	3.54 ± 0.21	3.46 ± 0.96	6.29 ± 1.01	< 0.001	0.84	< 0.001	< 0.001
6th hr	3.34 ± 0.78	3.26 ± 0.59	6.46 ± 1.01	< 0.001	0.81	< 0.001	< 0.001
12th hr	3.57 ± 0.23	3.34 ± 0.83	6.77 ± 0.73	< 0.001	0.56	< 0.001	< 0.001
24th hr	3.34 ± 0.86	2.97 ± 0.79	5.83 ± 0.66	< 0.001	0.31	< 0.001	< 0.001
**VAS (at activity)**							
Immediately	3.80 ± 0.23	3.11 ± 0.65	6.46 ± 0.95	< 0.001	0.30	< 0.001	< 0.001
2nd hr	4.06 ± 0.71	3.40 ± 0.71	7.86 ± 0.81	< 0.001	0.06	< 0.001	< 0.001
4th hr	4 ± 2	3.46 ± 0.96	6.89 ± 0.58	< 0.001	0.17	< 0.001	< 0.001
6th hr	3.83 ± 0.54	3.31 ± 0.67	6.74 ± 0.70	< 0.001	0.12	< 0.001	< 0.001
12th hr	4.23 ± 0.52	3.43 ± 0.96	6.89 ± 0.67	< 0.001	0.02	< 0.001	< 0.001
24th hr	3.86 ± 0.59	3 ± 0.83	6.23 ± 0.77	< 0.001	0.01	< 0.001	< 0.001

Data expressed as mean (SD), number (percentage). *P* value was significant if < 0.05. **ESP**: erector spinae plane block; **TPV**: thoracic paravertebral block; **VAS**: visual analogue scale

***P*value** compares between different groups

***P*1** compares between ESP and TPV groups

***P*2** compares between ESP mg and control groups

***P*3** compares between TPV mg and control groups

*Analysis was done by ANOVA test followed by post-hoc analysis

#### Secondary outcome

The ESP and TPV groups were associated with a longer time period before the first analgesic request in comparison to the control group (*p* < 0.001). This time period was of shorter duration in the ESP group than that in the TPV group (*p* = 0.03). (Table 2)

The ESP and TPV groups had lower postoperative VAS scores at different times of assessment (*p* < 0.001), either at rest or during movement, in comparison to the control group. The ESP group obtained higher VAS scores during activity at 12 and 24 h postoperatively in comparison to the TPV block (*p* > 0.05) (Table 2).

**Fig. 4:**
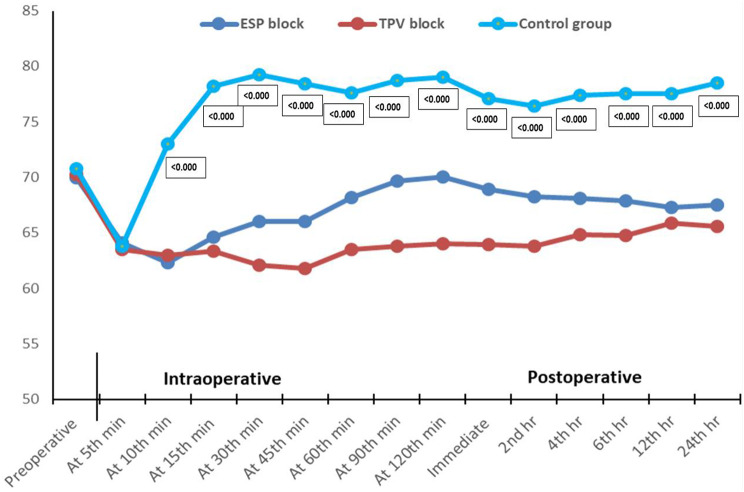
Perioperative assessment of MAP among the studied groups. ESP: erector spinae plane block; TPV: thoracic paravertebral block; MAP: mean arterial blood pressure

Furthermore, the ESP and TPV groups had insignificant differences as regards mean arterial blood pressure (MAP) from the baseline till the 30th intraoperative minute (*p* > 0.05). Then, the ESP group had a higher MAP till the 6th postoperative hour (*p* < 0.05), although the difference was not statistically significant (*p* > 0.05). Overall, MAP was lower in the case of the ESP and TPV groups (*p* < 0.001), in comparison to the control group, at different times of assessment except for baseline and the 5th intraoperative minute, where both groups had a comparable MAP (*p* > 0.05) (Fig. 4).

**Fig. 5:**
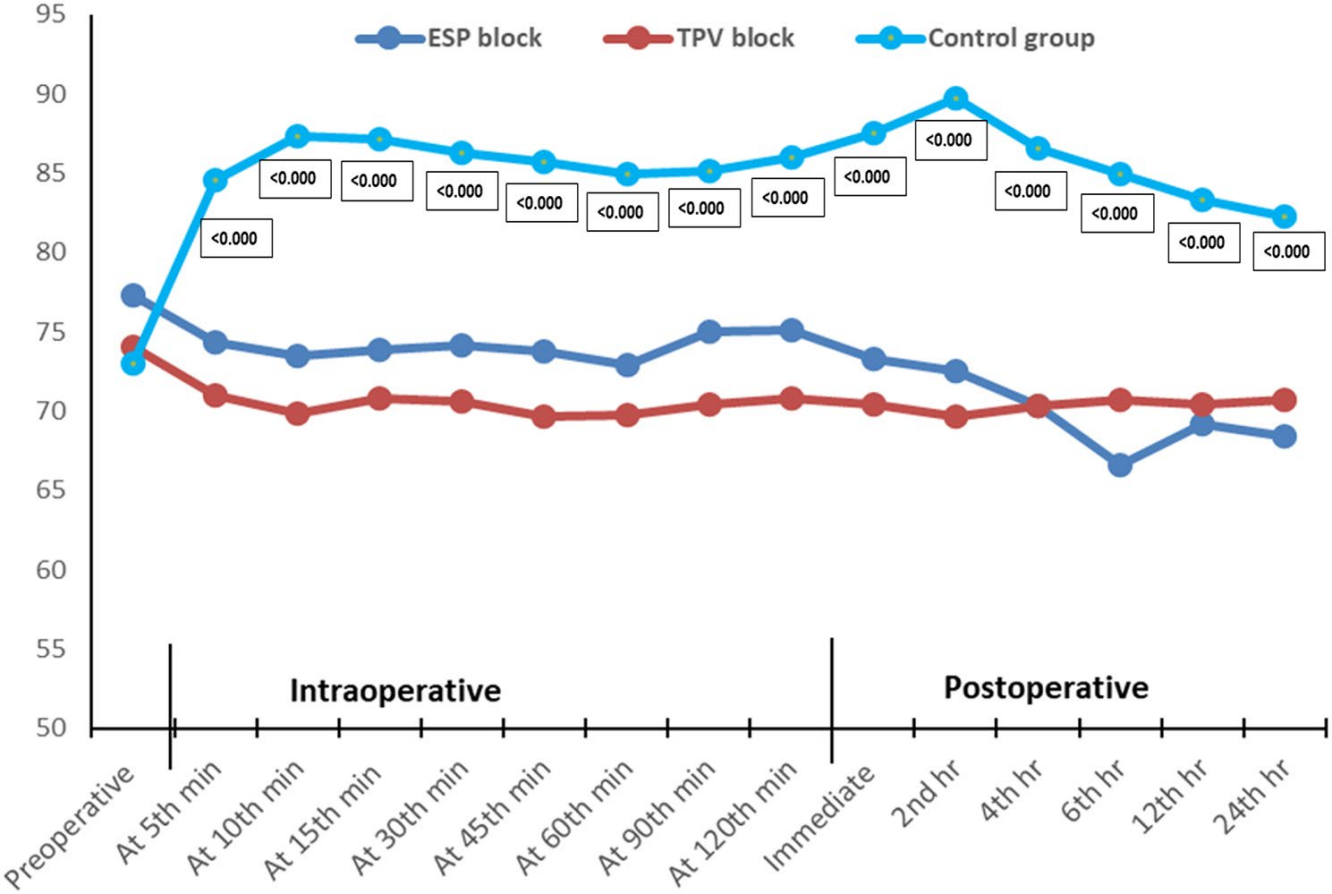
Perioperative assessment of HR among the studied groups. ESP: erector spinae plane block; TPV: thoracic paravertebral block; HR: heart rate

The ESP and TPV groups exhibited insignificant differences as regards heart rate (HR) from baseline till the 15th intraoperative minute (*p* > 0.05). Thereafter, ESP block was associated with a higher HR till immediately after surgery (*p* < 0.05) when both groups demonstrated comparable HRs (*p* > 0.05). HRs were lower in the case of the ESP and TPV groups (*p* < 0.001) in comparison to the control group at different times of assessment, except for baseline when both groups had comparable HRs (*p* > 0.05) (Fig. 5).

Regarding sedation levels, the ESP and TPV groups exhibited higher sedation in comparison to the control group immediately after surgery, two, and four hours postoperatively. No significant differences were observed in this regard between the TPV and ESP groups.

The ESP and TPV groups expressed better postoperative satisfaction levels at different times of assessment (*p* < 0.001) in comparison to the control group. However, the ESP group had a lower satisfaction level at the 6th postoperative hour in comparison to the TPV group (*p* = 0.01) (Table 3).

**Table 3:** Postoperative satisfaction and sedation level among the studied groups

Satisfaction level	ESP bock group (*n* = 35)	TPV block group (*n* = 35)	Control group (*n* = 35)	*P* value*	Subgroup analysis		
					P1	P2	P3
**Post-operative**							
Immediate	2.63 ± 0.65	2.63 ± 0.73	1.71 ± 0.45	< 0.001	1.00	0.01	< 0.001
2nd hr	2.97 ± 0.89	3.23 ± 0.91	2.34 ± 0.48	< 0.001	0.17	< 0.001	< 0.001
4th hr	2.74 ± 0.89	2.91 ± 0.70	2.40 ± 0.50	< 0.001	0.31	0.04	< 0.001
6th hr	2.89 ± 0.80	3.34 ± 0.99	2.37 ± 0.55	< 0.001	0.01	< 0.001	< 0.001
12th hr	2.69 ± 0.76	2.83 ± 0.62	2.26 ± 0.44	< 0.001	0.33	< 0.001	< 0.001
24th hr	2.74 ± 0.51	2.83 ± 0.45	2.31 ± 0.47	< 0.001	0.45	< 0.001	< 0.001
**Sedation level**				***P*** value*	**Subgroup analysis**		
					***P*** **1**	***P*** **2**	***P*** **3**
**Post-operative**							
Immediate	4.29 ± 0.67	3.86 ± 0.91	4.74 ± 0.78	< 0.001	0.02	0.01	< 0.001
2nd hr	4.97 ± 0.17	5	5	0.37	0.22	0.22	1.00
4th hr	5	5	5	---	---	---	---
6th hr	5	5	5	---	---	---	---
12th hr	5	5	5	---	---	---	---
24th hr	5	5	5	---	---	---	---

Data expressed as mean (SD). *P* value was significant if < 0.05. **ESP**: erector spinae plane block; **TPV**: thoracic paravertebral block

***P*value** compares between different groups

***P*1** compares between ESP and TPV groups

***P*2** compares between ESP mg and control groups

***P*3** compares between TPV mg and control groups

*Analysis was done by ANOVA test followed by post-hoc analysis

Furthermore, the ESP and TPV groups were associated with lower perioperative cortisol and prolactin levels at different times of assessment in comparison to the control group (*p* < 0.001). The differences between the ESP and TPV groups were not statistically significant for these factors (*p* > 0.05) (Table 4).

**Table 4:** Perioperative assessment of cortisone and prolactin level among the studied groups

	ESP bock group (*n* = 35)	TPV block group (*n* = 35)	Control group (*n* = 35)	*P* value*	Subgroup analysis		
					P1	P2	P3
**Cortisol level (mcg/dl)**							
Preoperative (T0)	14.28 ± 2.33	14.81 ± 2.62	12.72 ± 2.89	0.14	0.63	0.15	0.06
After intubation (T1)	21.51 ± 6.75	19.17 ± 6.27	29.11 ± 9.28	< 0.001	0.19	< 0.001	< 0.001
Immediately postoperative (T2)	21.84 ± 11	20.67 ± 9.59	34.59 ± 9.36	< 0.001	0.62	< 0.001	< 0.001
24th postoperative hour (T3)	22.98 ± 14.08	23.03 ± 5.16	34.81 ± 9.67	< 0.001	0.98	< 0.001	< 0.001
**Prolactin level (ng/ml)**							
Preoperative (T0)	11.08 ± 2.92	11.54 ± 1.89	11.01 ± 2.77	0.88	0.69	0.95	0.65
After intubation (T1)	17.48 ± 4.19	18.33 ± 2.57	25.09 ± 2.14	< 0.001	0.74	< 0.001	< 0.001
Immediately postoperative (T2)	18.84 ± 2.81	20.94 ± 3.87	30.34 ± 2.08	< 0.001	0.41	< 0.001	< 0.001
24th postoperative hour (T3)	20.29 ± 4.94	22.25 ± 5.30	35.28 ± 9.51	< 0.001	0.54	< 0.001	< 0.001

Data expressed as mean (SD). *P* value was significant if < 0.05. **ESP**: erector spinae plane block; **TPV**: thoracic paravertebral block

***P*value** compares between different groups

***P*1** compares between ESP and TPV groups

***P*2** compares between ESP mg and control groups

***P*3** compares between TPV mg and control groups

*Analysis was done by ANOVA test followed by post-hoc analysis

**Fig. 6:**
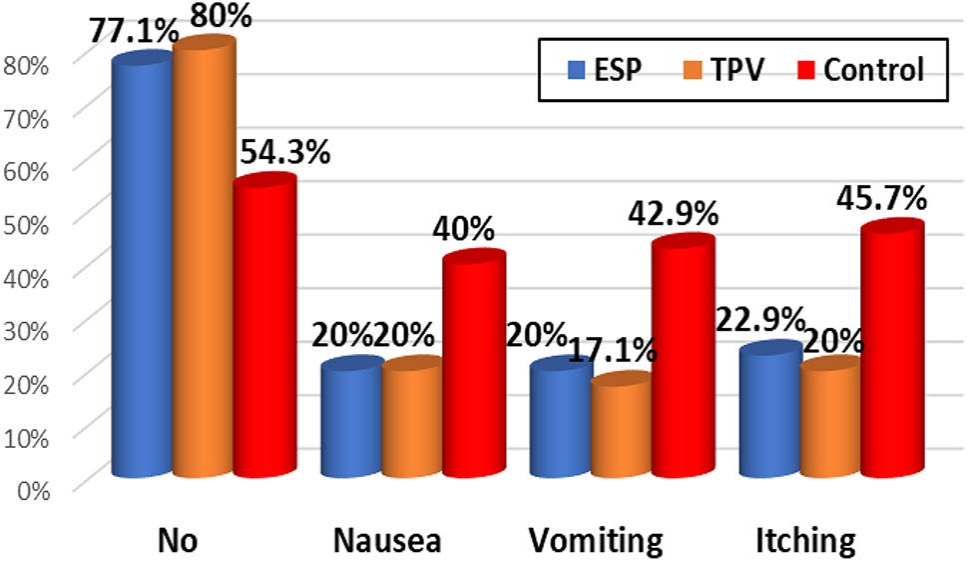
Post-operative side effects among the studied groups. ESP: erector spinae plane block; TPV: thoracic paravertebral block

The incidence of side effects in the studied groups was higher in the control group than those in the other groups with regard to the occurrence of vomiting, nausea, and itching (*p* = 0.029, *p* = 0.092, *p* = 0.036, respectively) (Fig. 6).

The LANSS scores of the ESP and TPV groups were lower in the 1st, 3rd, and 6th postoperative months (*p* < 0.05) in comparison to the control group. The differences between the scores of the ESP and TPV groups were not statistically significant (*p* > 0.05). As regards the frequency of chronic pain in the different groups, 7 (20%), 6 (17.1%), and 10 (28.6%) patients in the ESB, TPV, and control groups, respectively, suffered from chronic pain at different times of the assessment (Table 5).

**Table 5:** Postoperative assessment of LANSS score in the studied groups

	ESP bock group (*n* = 35)	TPV block group (*n* = 35)	Control group (*n* = 35)	*P* value*	Subgroup analysis		
					P1	P2	P3
**LANSS**							
1st month	8.91 ± 3.72	7.26 ± 3.76	10.17 ± 3.36	0.009	0.06	0.04	< 0.001
3rd month	8.80 ± 4.05	7.77 ± 3.78	10.89 ± 4.56	0.004	0.30	0.03	0.002
6th month	8.23 ± 4.33	8.06 ± 2.32	11.60 ± 3.19	0.002	0.87	0.002	0.003

Data expressed as mean (SD). *P* value was significant if < 0.05. **ESP**: erector spinae plane block; **TPV**: thoracic paravertebral block

***P*value** compares between different groups

***P*1** compares between ESP and TPV groups

***P*2** compares between ESP mg and control groups

***P*3** compares between TPV mg and control groups

*Analysis was done by ANOVA test followed by post-hoc analysis

## Discussion

The purpose of this randomized comparative trial was to evaluate the impact of the ultrasound-guided thoracic paravertebral block (TPV) and erector spinae plane block (ESP) on stress response as well as acute and chronic post-mastectomy pain after a modified radical mastectomy. The results of our study demonstrated that ESP and TPV blocks provided superior early postoperative analgesia measured by total morphine consumption in postoperative 24 h, time of first request of analgesia, VAS score, and reduced stress response compared to the control group in female patients undergoing modified radical mastectomy without serious side effect. Both techniques showed better long-term outcomes compared to the control group regarding LANSS score in 6 months follow-up.

According to GLOBOCAN 2020 estimates, there are an expected 2.3 million new cases of breast cancer globally, making it one of the most common malignancies and the fifth leading cause of cancer-related deaths. For individuals undergoing major procedures for breast cancer, post-mastectomy pain syndrome (PMPS) can be an agonizing illness that results in long-term impairment [15].

Moreover, it is possible to reduce the onset and intensity of chronic pain by using a localized anesthetic. Less central sensitization (wind-up) and opioid-induced hyperalgesia are two suggested methods for lowering chronic pain [16]. During TPV block, there is the anteromedial spread of local anesthetic into the paravertebral space combined with lateral intercostal spread. The ventral rami of the spinal nerve and the sympathetic ganglion are usually involved in a successful TPV block, and epidural spread through the intervertebral foramen is often noted [17]. ESP block ends at the transverse process of the spine and poses no risk to any vital structures along the needling path, making it safe and easy to perform. Anesthetic coverage close to that of the TPV block is achieved using the ESP block. The recent finding of two slits at the medial and lateral extremities of the superior costo-transverse ligament that can function as conduits to the thoracic paravertebral region supports this theory. During ESP block, there is a significant spread of the local anesthetic in the fascial layer and the back muscles [18].

We showed in our study that total analgesic consumption, time to first analgesic request, and VAS (either at rest or activity) varied significantly across groups. Compared to the TPV block group, the ESP block group required a significantly higher total amount of analgesic while having a significantly shorter time until the first analgesic request had a considerably greater VAS during activity on the 12th and 24th postoperative hours. Both groups showed considerably lower total morphine consumption than the control group according to Elewa et al. [19], though patients in the ESP block group consumed morphine at an insignificantly decreased rate compared to the TPV block group despite of having the same randomization of the three groups. However, El Ghamry et al. [20] reported no differences between TPV and ESP block groups in terms of total analgesic use, time to initial rescue analgesia, or pain ratings after breast surgeries. But they randomized the patients who were enrolled in their study into just two groups of patients comparing between ESP and PVB only without control group. Additionally, Gürkan et al. [21] reported no difference between the ESP and TPV block groups regarding 24-h morphine consumption, despite having the same methodology of our trial, and there were differences between both the ESP and TPV block groups compared with the control groups.

Our results are consistent with those of a recent randomized double-blind trial conducted by Swisher et al. [22], which showed that TPV block resulted in lower VAS ratings and less morphine intake than ESP block in the first 24 h following mastectomy surgery. But they used ropivacaine 0.5% with epinephrine in both blocks. According to a recent randomized double-blind trial by Wittayapairoj et al. [23], the TPV block group had lower overall NRS and 24-h total morphine consumption than the ESP block group. We postulated that the greater analgesic impact of TPV block might be caused by less direct distribution of anesthetic to the paravertebral area after ESP block compared to adequate dissemination after TPV block. In another study, Santonastaso et al. [24], demonstrated that the use of single shot ultrasound guided thoracic paravertebral block alone or combined with general anaesthesia for modified radical mastectomy, led to avoidance of use of opioids either intraoperatively or postoperatively.

Our study revealed that there were no significant differences in mean arterial pressure (MAP) between the ESP and TPV block groups within the first 30 intraoperative minutes; after that, the ESP block group had a higher MAP until the sixth postoperative hour, following which the MAPs of the two block groups became comparable. Moreover, we observed that MAP in the ESP and TPV block groups was lower than in the control group, except at baseline and the fifth intraoperative minute when the MAPs of the three groups were comparable. From the baseline until the 15th intraoperative minute, there were no significant differences in HR between the ESP and TPV block groups; afterward, the ESP block group had a higher HR until the early postoperative period, after which the HRs of the two block groups became comparable. The HRs of the ESP and TPV block groups were lower than that of the control group, except for baseline when HRs were comparable.

In contrast to Elewa et al. [19], the mean heart rate did not substantially change between the analyzed groups at baseline, 5, 10, 15, 20, 25, 30, 45, 75, 90, 105, or 120 min throughout the operation; however, at 60 min post-op, the mean heart rate was considerably lower in the ESP and TPV block groups than in the control group. During the baseline, 5, 10, 20, 25, 30, 45, 60, 75, 90, 105, and 120-min periods, the MAP did not substantially change among the groups; however, 15 min after the procedure, the MAP was considerably lower in the ESP block group than in the control group. Insignificant differences were observed in cortisol and prolactin levels after intubation between ESP and TPV blocks immediately following surgery as well as 24 h after surgery, though levels in both groups were significantly lower than those in the control group. Our findings correlate with those of Lin et al. [25], who reported that patients who had radical mastectomy had significantly lower cortisol levels immediately following intubation and extubation with TPV block compared to the control group. However, they also found in their study that no significant difference in the levels of adrenocorticotropic hormone (ACTH) between the two groups immediately after tracheal intubation, and the levels of ACTH in the study group were significantly lower than the control group. It worth mentioning that, they used a ultrasound-guided continuous thoracic paravertebral nerve block in patients undergoing radical mastectomy. Serum cortisone levels at 2 h after surgical incision, 2 h postoperatively, and 24 h postoperatively were considerably lower in the TPV block group than in the thoracic epidural group in patients undergoing elective thoracotomy not in patients undergo modified radical mastectomy as in our study, according to study results by Abd El-Hamid et al. [26]. It worth mentioning that they didn’t enrol ESP in their study opposite to our study.

The ability to lower postoperative opioid consumption and the subsequent risk of complications (such as postoperative nausea and vomiting) is the main advantage of preoperative nerve block procedures. In contrast to general anesthesia alone, our study observed that ESP and TPV blocks reduced the incidence of postoperative nausea, vomiting, and itching. These results agree with those of a prior randomized control trial conducted by Elewa et al. [19]. Using the LANSS score, we demonstrated that both TPV and ESP block reduced the incidence of chronic post-mastectomy pain compared to the control group. The concept that TPVB significantly lowers pain levels during the early postoperative phase and boosts patient satisfaction is supported by a number of research; [27, 28] moreover, TPVB may lessen chronic pain [29, 30]. Compare the effectiveness of the thoracic paravertebral block and the erector spinae plane block in radical mastectomy surgeries, with or without axillary emptying, as reported by Santonastaso and colleagues [31]. They discovered that throughout the six-month follow-up period following surgery, no patients had complained of chronic pain.

### Study limitations and future studies

As a limitation of this study, the dermatomal distribution of these two blocks may be ascertained by sensory testing. For future research, it would be preferable to display and contrast the precise boundaries of the blocks.

The second limitation was the investigator who performed the blocks wasn’t blinded to the group, which could influence the outcomes and the third one was that the nerve block was performed with a single injection rather than a catheter. Also, the fact that this study is single-center is another drawback.

## Conclusion

ESP and TPV blocks provided superior early postoperative analgesia and reduced stress response compared to the control group in female patients undergoing modified radical mastectomy. PVB is better than ESB in acute postoperative pain management (the total morphine consumption VAS score and time of first analgesic request). Both techniques showed better long-term outcomes compared to the control group regarding LANSS score in the 6-month follow-up.

## Electronic supplementary material

Below is the link to the electronic supplementary material.

Supplementary Material 1

## Data Availability

The database is closed and there is no public access. However, permission to access and use the database can be obtained if necessary by request the corresponding author.

## Abbreviations

ESPB: Erector Spinae plan Block
TPVB: Thoracic Paravertebral block
PMPS: Postmastectomy pain syndrome
LA: Local anaesthetics
ASA: American Society of Anaesthesiology physical status classification
MAP: Mean arterial blood pressure
HR: Heart rate
VAS: Visual Analogue Score
MOAA/S scale: Modified Observer’s Assessment of Alertness/Sedation Scale
LANSS score: Leeds Assessment of Neuropathic symptoms and signs score
P.C.A: Patient controlled analgesia
U/S: Ultrasound
SPSS: Statistical Package for the Social Science
PEC I and II: Pectoral nerve blocks I and Pectoral nerve blocks II
SAP: Serratus anterior plane block
SCTL: Superior costo-transverse ligament
