# Supplementary material for: Comparison between ultrasound guided erector spinae plane block and paravertebral block on acute and chronic post mastectomy pain after modified radical mastectomy: randomized controlled trial
Source: BMC Anesthesiol. 2024 Nov 21;24:420. doi: 10.1186/s12871-024-02810-4 (PMC11580581; doi:10.1186/s12871-024-02810-4)
Supplement: Supplementary file 1 — Supplementary Material 1 [file 12871_2024_2810_MOESM1_ESM.docx]

1-Visual Analouge Scale :


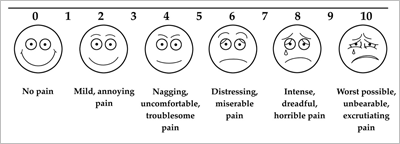


1-3 : Mild pain 4-6 : moderate pain 7-10 : severe pain

2-Modified Observer's Assessment of Alertness/Sedation Scale (MOSS/A).

| Responsivness | Score |
| --- | --- |
| Agitaed | 6 |
| Responds Readialy to name spoken in normal tone | 5 |
| Lethargic response to name spoked in normal tone | 4 |
| Responds only after name is called loudly and/or repeatedly | 3 |
| Responds only after mild prodding or shaking | 2 |
| Doesnot respond to mild prodding or shaking | 1 |
| Doesnot respond to deep stimulus | 0 |

3- Pateint satisfaction score :

| Level of satisfaction | Score |
| --- | --- |
| Extremely excellent | 4 |
| Being good | 3 |
| Being fair | 2 |
| Being bad | 1 |

4-LANSS Scale. Leeds Assessment of Neuropathic Symptoms and Signs (LANSS) Pain Scale (Bennett, 2001)

**A. Pain Questionnaire**

1. Does your pain feel like strange, unpleasant sensations in your skin? Words like pricking, tingling, pins and needles might describe these sensations.

a) NO – My pain doesn’t really feel like this ………………………………………………………. (0)

b) YES – I get these sensations quite a lot ………………………………………………………......(5)

2. Does your pain make the skin in the painful area look different from normal? Words like mottled or looking more red or pink might describe the appearance.

a) NO – My pain doesn’t affect the color of my skin ……………………………………………... (0)

b) YES – I’ve noticed that the pain does make my skin look different from normal ………………(5)

3. Does your pain make the affected skin abnormally sensitive to touch? Getting unpleasant sensations when lightly stroking the skin, or getting pain when wearing tight clothes might describe the abnormal sensitivity.

a) NO – My pain doesn’t make my skin abnormally sensitive in that area ………………………..(0) b) YES – My skin seems abnormally sensitive to touch in that area................................................(3)

4. Does your pain come on suddenly and in bursts for no apparent reason when you’re still? Words like electric shocks, jumping and bursting describe these sensations.

a) NO – My pain doesn’t really feel like this………………………………………………………. (0)

b) YES – I get these sensations quite a lot ………………………………………………………….(2)

5. Does your pain feel as if the skin temperature in the painful area has changed abnormally? Words like hot and burning describe these sensations.

a) NO – I don’t really get these sensations……………………………………………………… (0)

b) YES – I get these sensations quite a lot ………………………………………………………(1)

**B. Sensory Testing**

Skin sensitivity can be examined by comparing the painful area with a contralateral or adjacent nonpainful area for the presence of allodynia and an altered pin-prick threshold (PPT).

**1. Allodynia**

Examine the response to lightly stroking cotton wool across the non-painful area and then the painful area. If normal sensations are experienced in the non-painful site, but pain or unpleasant sensations (tingling, nausea) are experienced in the painful area when stroking, allodynia is present.

a) NO – Normal sensations in both areas…………………………………………………………(0)

b) YES – Allodynia in painful area only………………………………………………………… (5)

**2. Altered pin-prick threshold**

Determine the pin-prick threshold by comparing the response to a 23-gauge needle mounted inside a 2ml syringe barrel placed gently onto the skin in a non-painful and then painful areas. If a sharp pin prick is felt in the non-painful area, but a different sensation is experienced in the painful area, e.g. none/ blunt only (raised PPT) or a very painful sensation (lowered PPT), an altered PPT is present. If a pinprick is not felt in either area, mount the syringe onto the needle to increase the weight and repeat.

a) NO – Equal sensation in both areas……………………………………………………………(0)

b) YES – Altered PPT in painful area……………………………………………………………(3)

Total score ………………………………………………………………………………Maximum 24

If score ˂12 , neuropathic mechanism are **unlikely** to be contributing to the patient pain

If score ˃ 12 , neuropathic mechanism are **likely** to be contributing to the patient pain
